# Structure of a cyclin-dependent kinase from *Giardia lamblia*

**DOI:** 10.1107/S1744309111018070

**Published:** 2011-08-16

**Authors:** David J. Leibly, Paul A. Newling, Jan Abendroth, Wenjin Guo, Angela Kelley, Lance J. Stewart, Wesley Van Voorhis

**Affiliations:** a Seattle Structural Genomics Center for Infectious Disease (SSGCID), USA; b Department of Medicine, Division of Allergy and Infectious Diseases, School of Medicine, University of Washington, Box 356423, Seattle, WA 98195-6423, USA; c Emerald BioStructures Inc., 7869 NE Day Road West, Bainbridge Island, WA 98110, USA; d Seattle Biomed, 307 Westlake Avenue North, Suite 500, Seattle, WA 98109, USA

**Keywords:** *Giardia lamblia*, cyclin-dependent kinases, cyclin, giardiasis, adenosine monophosphate, SSGCID

## Abstract

*Giardia lamblia* is the etiologic agent of giardiasis, a water-borne infection that is prevalent throughout the world. The need for new therapeutics for the treatment of giardiasis is of paramount importance. Owing to the ubiquitous nature of kinases and their vital importance in organisms, they are potential drug targets. In this paper, the first structure of a cyclin-dependent kinase (CDK) from *G. lamblia* (*Gl*CDK; UniProt A8BZ95) is presented. CDKs are cell-cycle-associated kinases that are actively being pursued as targets for anticancer drugs as well as for antiparasitic chemotherapy. Generally, a CDK forms a complex with its associated cyclin. This CDK–cyclin complex is active and acts as a serine/threonine protein kinase. Typically, CDKs are responsible for the transition to the next phase of the cell cycle. Although the structure of *Gl*CDK with its associated cyclin was not solved, the 1.85 Å resolution structure of apo *Gl*CDK and a 2.0 Å resolution structure of *Gl*CDK in complex with adenosine monophosphate are presented and the structural differences from the orthologous human CDK2 and CDK3 are discussed.

## Abstract 2

Crystal structures of a cyclin-dependent kinase from *G. lamblia* are presented in both apo and AMP-bound forms.

## 1. Introduction

### 1.1. *Giardia lamblia*

*G. lamblia* (also referred to as *G. duodenalis* or *G. intestinalis*) is a water-borne flagellated parasite that causes giardiasis in humans. Host-to-host transference of *Giardia* cysts takes place *via* the fecal–oral route (Dawson & House, 2010 ▶). This allows the protozoan to infect the next generation of hosts through a diverse range of mechanisms: person to person, animal to human or by contact with contaminated water and food (Plutzer *et al.*, 2010 ▶). Outbreaks commonly occur in areas with inadequate water treatment, especially developing countries, where infection rates can be higher than 50% of the total population (World Health Organization, 1992 ▶). *Giardia* is one of the leading protozoan causes of gastrointestinal illness worldwide and has joined the ranks of the WHO Neglected Diseases Initiative (Flanagan, 1992 ▶; Savioli *et al.*, 2006 ▶).

*G. lamblia* has a two-stage lifecycle: the cyst and the trophozoite. The cyst is able to pass intact through the stomach and excysts in the small intestine. The trophozite binds to the upper intestine, causing malabsorption and the symptoms of giardiasis (Birkeland *et al.*, 2010 ▶). Giardiasis in children, who are at a higher risk of infection, is associated with a failure to thrive and impaired cognitive function and can lead to death (Savioli *et al.*, 2006 ▶; Centers for Disease Control and Prevention, 2010 ▶).

The current treatment options for *Giardia* are limited. Furazolidone and quinacrine have availability problems and metronidazole and paromomycin are not FDA-approved for the treatment of giardiasis in the US (The Medical Letter, 2010 ▶). There is also concern that resistance is emerging to the most commonly used therapeutic, metronidazole (Upcroft *et al.*, 2009 ▶). It is clear with *G. lamblia* being such a ubiquitous pathogen that new drug therapies are of the utmost importance to the global community.

### 1.2. Cyclin-dependent kinases

Cyclin-dependent kinases (CDKs) are typically described as regulators of the cell cycle. Different CDKs become activated *via* their cyclin partners at different times during cell-cycle progression (Vermeulen *et al.*, 2003 ▶). A CDK inhibitor would lock the cell cycle in place and prevent growth, which is the precise reason why CDK inhibitors are under clinical trial for various types of cancer (Rossi *et al.*, 2006 ▶).

CDKs have not only been found in humans and higher-level organisms but also in lower-level organisms such as protozoa (Naula *et al.*, 2005 ▶). Thus, the use of CDK inhibitors could have far-reaching implications. CDK inhibitors have previously been shown to accelerate apoptosis in mammalian cells (Rossi *et al.*, 2006 ▶). As mentioned above, human CDK inhibitors are currently receiving an influx of pharmaceutical investment for oncological indications. The CDK from *G. lamblia* (*Gl*CDK) has two human homologues with similar overall amino-acid identities of over 50%, with the other human homologues all having above 33% similarity (Table 1 ▶). All of the human CDKs presented in Table 1 ▶ have been structurally elucidated, with the exception of human CDK3. Given that human CDKs can be inhibited, leading to apoptosis, *Gl*CDK may also be a potential drug target for the treatment of giardiasis. The lack of significant overall amino-acid sequence identity to human CDKs indicates that it may be possible to selectively inhibit the *Giardia* protein. The structural investigations reported here were performed in order to further investigate *Gl*CDK as a drug target.

## 2. Methods

### 2.1. Protein expression and purification

The gene encoding the CDK from *G. lamblia* (*Gl*CDK; UniProt A8BZ95) was PCR-amplified in a 96-well format using genomic DNA as a template. The primers were designed with an additional ligation-independent cloning (LIC; Aslanidis & de Jong, 1990 ▶) sequence at their 5′ ends that was complementary to the LIC sequence in the plasmid vector. The purified PCR products were then cloned *via* LIC into the AVA0421 expression vector (Quartley *et al.*, 2009 ▶), which provided a C3-cleavable six-histidine (His_6_) tag at the N-­terminus of the expressed protein with sequence MAHHHHHHMGTLEAQTQ/GPGS (the protease cleavage site is indicated by a slash). The recombinant plasmid was then transformed into *Escherichia coli* Rosetta Oxford strain [BL21*(DE3)-R3-pRARE2] cells for expression testing (Mehlin *et al.*, 2006 ▶; Choi *et al.*, 2011 ▶).

A starter culture of LB broth with appropriate antibiotics was grown for ∼18 h at 310 K. ZYP-5052 auto-induction medium was freshly prepared as per UW-PPG protocols (Studier, 2005 ▶; Choi *et al.*, 2011 ▶). Antibiotics were added to 2 l bottles of sterile auto-induction medium. The bottles were inoculated with all of the overnight culture. The inoculated bottles were then placed into a LEX Bioreactor (Harbinger Biotechnology, Toronto, Ontario). Cultures were grown for ∼24 h at 298 K; the temperature was then reduced to 288 K and the cultures were grown for a further ∼60 h. To harvest, the medium was centrifuged at 4000*g* for 20 min at 277 K. The cell paste was then flash-frozen in liquid nitrogen and stored at 193 K.

The frozen pellet was thawed and purified as per the SSGCID protocol (Bryan *et al.*, 2011 ▶). Briefly, the pellet was completely resuspended in lysis buffer (20 m*M* HEPES pH 7.2–7.4, 300 m*M* NaCl, 5% glycerol, 30 m*M* imidazole, 0.5% CHAPS, 10 m*M* MgCl_2_, 3 m*M* β-mercaptoethanol, 1.3 mg ml^−1^ protease-inhibitor cocktail and 0.05 mg ml^−1^ lysozyme). The resuspended cell pellet was then disrupted on ice for 15 min using a Branson Digital Sonifier 450D (set to 70% amplitude, with alternating cycles of 5 s pulse-on and 10 s pulse-off). The cell debris was incubated with 20 units ml^−1^ Benzon­ase nuclease (EMD Chemicals, Gibbstown, New Jersey, USA) at room temperature for at least 40 min and was then clarified by centrifugation with a Sorvall RC5 at 10 000 rev min^−1^ for 60 min at 277 K in a F14S Rotor (Thermo Fisher). The His_6_-tagged *Gl*CDK was separated from the clarified cell lysate by IMAC on a HisTrap FF 5 ml column (GE Biosciences, Piscataway, New Jersey, USA) equilibrated with binding buffer (20 m*M* HEPES pH 7.0, 300 m*M* NaCl, 5% glycerol, 30 m*M* imidazole, 1 m*M* TCEP). The recombinant *Gl*CDK was eluted in wash buffer by the addition of 500 m*M* imidazole with a step elution and was further resolved by size-exclusion gel chromatography (SEC; Superdex 75 26/60, GE Biosciences). The His_6_ affinity tag was not removed from the protein. Pure fractions collected in SEC buffer (20 m*M* HEPES pH 7.0, 300 m*M* NaCl, 2 m*M* DTT and 5% glycerol) as a single peak were analyzed using SDS–PAGE and Simply Blue Safestain (Invitrogen Corp, Carlsbad, California, USA). The protein was then pooled, concentrated to 27.3 mg ml^−1^, flash-frozen and stored at 193 K in SEC buffer. The purified protein in this final buffer was used in the crystallization trials.

### 2.2. Crystallization

Two sparse-matrix screens were set up with purified *Gl*CDK at a final concentration of 25.2 mg ml^−1^ using the JCSG+, Cryo (Emerald BioStructures, Bainbridge Island, Washington, USA), PACT and ProPlex (Molecular Dimensions, Apopka, Florida, USA) screens following an extended Newman’s strategy (Newman *et al.*, 2005 ▶). 0.4 µl protein solution was mixed with 0.4 µl reservoir solution and equilibrated against 100 µl reservoir solution using 96-well Compact Jr Crystallization plates (Emerald BioStructures). Crystals were obtained in several PEG-containing conditions. Crystals that were suitable for diffraction studies were found in PACT screen condition F9: 200 m*M* sodium/potassium tartrate, 100 m*M* Bis-Tris propane pH 6.5, 20% PEG 3350. Native *Gl*CDK crystals were cryoprotected by short soaks in buffers consisting of reservoir solution with an additional 10 and 20% glycerol and were then vitrified by plunging them into liquid nitrogen. For the ligand-bound structure, a crystal from the same drop was harvested in a soaking buffer consisting of 200 m*M* sodium/potassium tartrate, 100 m*M* Bis-Tris propane, 25% PEG 3350, 1 m*M* adenosine monophosphate (AMP) and 1 m*M* MgCl_2_. After an overnight soak, the crystal was vitrified as before by plunging it directly into liquid nitrogen.

### 2.3. Data collection and structure determination

Data sets were collected in-house from native and AMP-bound crystals at the Cu *K*α wavelength using a Rigaku SuperBright FR-E^+^ rotating-anode X-ray generator equipped with Osmic VariMax HF optics and a Saturn 944+ CCD detector (Table 2 ▶). For each data set, 720 images were collected with a fine ϕ-slicing of 0.25° per image. The diffraction data were reduced in space group *P*2_1_2_1_2_1_ using *XDS*/*XSCALE* (Kabsch, 2010 ▶) to 1.85 Å resolution.

The packing density (Matthews, 1968 ▶) suggested the presence of one *Gl*CDK molecule per asymmetric unit, with a *V*_M_ of 1.93 Å^3^ Da^−1^ and 36% solvent content. A search of the PDB for sequence homology yielded human cyclin-dependent kinase CDK2 (PDB entry 1oit; Anderson *et al.*, 2003 ▶) as the closest homolog, with 56% sequence identity. The search model was derived from monomer *A* of PDB entry 1oit using the *CCP*4 (Winn* et al.*, 2011 ▶) program *CHAINSAW* (Stein, 2008 ▶). Molecular replacement was performed with the *CCP*4 program *Phaser* (McCoy *et al.*, 2007 ▶) using data between 20 and 3.0 Å resolution; one molecule could be placed with high scores (RFZ = 12.5, TFZ = 17.7, LLG = 379, LLG_ref_ = 502). The model was then iteratively extended manually using *Coot* (Emsley *et al.*, 2010 ▶) followed by cycles of reciprocal-space refinement with the *CCP*4 program *REFMAC*5; H atoms were added in riding positions (Murshudov *et al.*, 2011 ▶).

**Figure 1:**
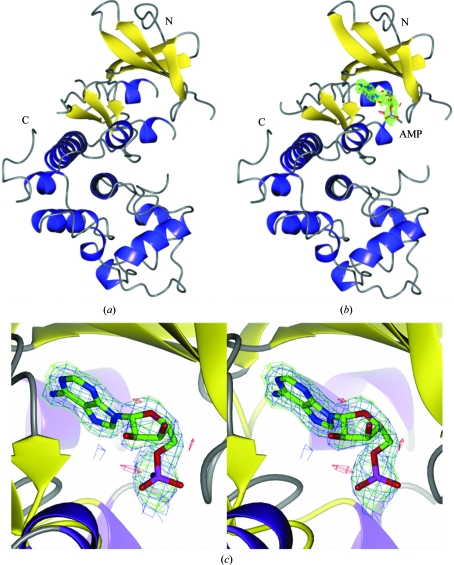
Structures of CDK kinase from *G. lamblia.* The apo structure (*a*) and the AMP-bound structure (*b*) are very similar. In (*b*) the OMIT *F*_o_ − *F*_c_ electron density for AMP is shown in green, contoured at 3.0σ. A close up of the OMIT density for AMP is shown in stereo representation in (*c*). The σ_A_-weighted electron-density maps are contoured at 1σ for the 2*F*_o_ − *F*_c_ map (blue, carved with a 2 Å radius around the AMP molecule) and at ±3σ for the *F*_o_ − *F*_c_ maps (green/red, carved with a 4 Å radius around the AMP molecule).

The native structure could be refined at 1.85 Å resolution to an *R*_work_ of 0.184 and an *R*_free_ of 0.236 with good stereochemistry (Table 3 ▶). The observed structure extended from residues Ser11 to Pro308 with three exceptions (Fig. 1 ▶ *a*). Three loops could not be modelled owing to weak electron density: Glu51–Thr58, Ser147–Glu151 and Ile168–Ile178. 209 water molecules were located. Four Ramachandran plot outliers were observed in *Coot*: Ile12 and Glu49, which are both adjacent to disordered regions, and Arg135 and Thr261, which both have well defined electron density.

The AMP-bound structure was solved by direct refinement of the apo structure after stripping off all solvent molecules. The structure was refined at 2.0 Å resolution to an *R*_work_ of 0.196 and an *R*_free_ of 0.265 with good stereochemistry (Table 3 ▶ and Fig. 1 ▶ *b*). The observed structure extended from residues Ser11 to Pro308, but again three loops could not be located owing to weak electron density: Glu51–Gly57, Ser147–Ser150 and Gly167–His175. 144 water molecules were modelled. Good OMIT density was observed for one AMP molecule (Fig. 1 ▶ *c*). The same Ramachandran plot outliers were observed as for the native protein, with the exception of Ile12.

The difference between *R*_cryst_ and *R*_free_ for the AMP-bound data set is 6.9%. This is higher than our usual goal in SSGCID for refinement (<5%). However, we do not see significant hints of over-refinement. The AMP-bound structure was re-refined with various numbers of TLS groups, which did not result in significant improvements in the *R* factors: *R*_cryst_/*R*_free_ were 0.196/0.265 for no TLS, 0.192/0.260 for one TLS group and 0.191/0.257 for five TLS groups (as suggested by *TLSMD*; Painter & Merritt, 2006 ▶). Most importantly, the maps of the three refinements were virtually identical. Owing to this, the deposited structures were not refined with TLS parameters. Additionally, we have observed hundreds of kinase structures through efforts by SSGCID and Emerald BioStructures and have found that the functional flexibility of the molecule is not properly described by the common refinement parameterization. This frequently renders high refinement *R* factors; nevertheless, the electron-density maps are of good quality.

## 3. Results and discussion

Full-length *Gl*CDK was purified with crystallizable quality under standard purification conditions. Diffraction-quality crystals were obtained without the need for optimization. Additionally, AMP was able to bind to the apo protein during an overnight soak.

**Figure 2:**
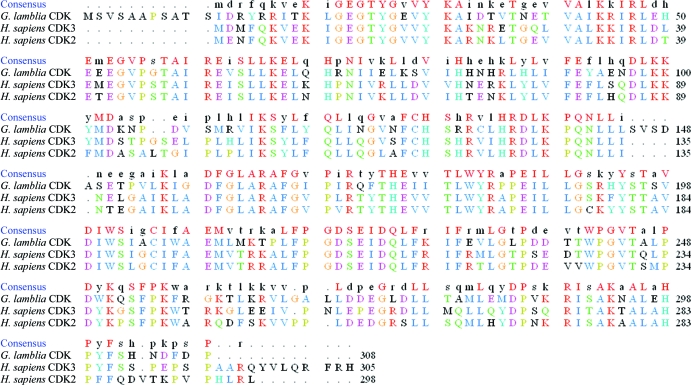
Amino-acid sequence alignment between *Gl*CDK, human CDK3 and human CDK2.

**Figure 3:**
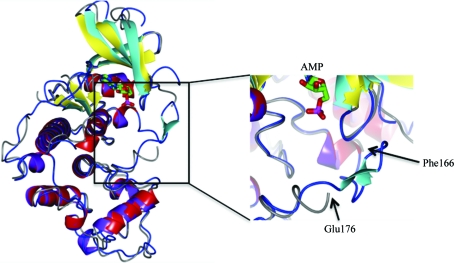
*Gl*CDK (PBD entry 3gc0) superposed with human CDK2 (PDB entry 1hcl; Schulze-Gahmen *et al.*, 1996 ▶). The *Giardia* protein is depicted in the same colours as in Fig. 1 ▶. CDK2 is depicted with cyan β-strands, red α-helices and blue loops. The insert depicts the different conformations of the activation loop in the two structures. Most of the activation loop is disordered in *Gl*CDK.

A 1.85 Å resolution apo data set was collected together with a 2.0 Å resolution data set for the AMP-bound protein using an in-house X-ray beam. The relatively high sequence identity to several human CDKs (Fig. 2 ▶) allowed determination of the *Gl*CDK structure by molecular replacement. The protein exhibited the expected fold for a CDK and existed as a monomer in the asymmetric unit (Fig. 3 ▶ *a*).

There is little structural variation between the apo and ligand-bound forms: 273 C^α^ atoms superimposed with an r.m.s.d. of 0.27 Å. Both structures contained three loops that could not be fully modelled owing to weak electron density. Residues Glu51–Thr58, Ser147–Glu151 and Ile168–Ile178 of the apo structure and residues Glu51–Gly57, Ser147–Ser150 and Gly167–His175 of the AMP-bound protein were not observed in our structures. However, the corresponding residues have been observed in several of the human CDK structures deposited in the PDB (see Fig. 3 ▶ *b*). NCBI annotation indicates that residues Gly158–Pro169 and Phe173–Trp181 are part of the kinase-activation loop. The weak electron density for residues Ile168–Ile178 of the apo structure and residues Gly167–His175 of the AMP-bound structure is consistent with flexibility of the kinase-activation loop. Fig. 3 ▶(*b*) depicts a superposition of *Gl*CDK and human CDK2. Although the activation loop is not fully observed in the *Gl*CDK structure, structural differences are apparent.

The unobserved activation loop contains the Thr174 residue that is phosphorylated in CDKs for full kinase activity (DeBondt *et al.*, 1993 ▶); human CDK1, CDK2 and CDK3 have the conserved sequence TFHEIVT which corresponds to Thr160–Thr167 of CDK2. Thr160 is the residue that is phosphorylated for full activity of CDK2 (DeBondt *et al.*, 1993 ▶). The homologous sequence of *Gl*CDK consists of TFH­EIIT (Thr174–Thr180). The inhibitory phosphorylation sites Thr25 and Tyr26, which correspond to residues Thr14 and Tyr15 of CDK2, are observed and are unphosphorylated in *Gl*CDK.

Furthermore, the unobserved loops Glu51–Thr58 (apo) and Glu51–Gly57 (AMP-bound) are predicted to be part of the CDK–cyclin interface that contains the residues Glu53–Val55 and Gly57 (NCBI annotation). This region corresponds to the PSTAIRE domain in human CDK (DeBondt *et al.*, 1993 ▶). The equivalent domain in *Gl*CDK consists of PGTAIRE (Pro56–Glu62). Pro56–Gly57 remain unobserved in the AMP-bound structure and in the apo structure. This sequence is conserved between the *Gl*CDK and human CDK structures, with differences in the structures of the preceding loops between the *Gl*CDK structure and the human CDK2 structure (PDB entry 1hcl; Schulze-Gahmen *et al.*, 1996 ▶).

The final conserved CDK regulatory GDSEID motif consists of Gly219–Asp224 of the *Giardia* protein. The amino-acid sequence is fully conserved between the *Giardia* and human proteins and there are no structural variations. Superposition of the ATP-binding site between *Gl*CDK and human CDK2 shows slight variations in the pocket but no significant structural variations (Schulze-Gahmen *et al.*, 1996 ▶).

A superposition of full-length *Gl*CDK and human CDK2 (Fig. 3 ▶) shows the expected structural homology of the proteins, with an r.m.s.d. of around 1.26 Å over 266 C^α^ atoms. Although the overall structure is very similar to those of the human CDKs, the sequence deviations in key regions observed in this study indicates that it may be possible to selectively inhibit *Gl*CDK. Given the large number of inhibitor-bound structures of human CDKs available in the PDB, it may be possible to model inhibitors in the *Gl*CDK active site. This can serve as a starting point for *Gl*CDK inhibitor development. The accessibility of the ATP-binding site to soaking suggests that similar soaks with ATP-competitive kinase inhibitors would be successful and could aid a structure-based drug-development approach. Given that human CDKs can be inhibited, leading to apoptosis, *Gl*CDK may also be a potential drug target for the therapy of giardiasis. Indeed, apoptotic cell death has been observed in a variety of protozoan pathogens, including *Giardia* (Chose *et al.*, 2003 ▶), and it seems likely that CDK inhibition would lead to apoptotic cell death in *Giardia*.

Further efforts need to be made to determine the native cyclin partner for the *Giardia* protein. To date, no kinase assays on this protein have been performed in our laboratory. It is unknown whether the cyclin is necessary for full activity in this case, although cyclins are generally required for full activity of CDKs. It is conceiv­able that cyclins from another species may be able to serve as a partner for activity assays or structure determination and this would enable the further development of inhibitors for *Gl*CDK without *de novo* protein production. The *Giardia* genome reveals at least six cyclins that have been annotated, including cyclin A and cyclin B homologs (http://giardiadb.org), and co-expression and testing with the CDK described here is feasible.

## 4. Conclusion

This paper describes the purification and structure determination of the first CDK from the pathogenic protozoon *G. lamblia*. The presented 1.85 Å resolution structure has structural homology to human CDK2 (PDB entry 1hcl) and a slightly higher sequence homology to human CDK3, which to date has no solved structure. This protein is from a class of validated drug targets; however, further work needs to be performed to determine whether the *Giardia* protein can be selectively inhibited over the human CDKs. To date, 25 *G. lamblia* protein structures have been deposited in the PDB. However, this is the first *Giardia* CDK structure to be determined.

## Supplementary Material

PDB reference: GilaA.00333.a, 3gbz

PDB reference: complex with AMP, 3gc0

## Figures and Tables

**Table 1:** Amino-acid homology between human CDKs and *G. lamblia* CDK

Human CDK	CDK3†	CDK2	CDK7	CDK6	CDK4	CDK9
Amino-acid sequence identity (%)	57.3	55.4	41.7	38.7	36.0	34.3

† The structure of CDK3 has not been elucidated.

**Table 2:** Data-collection statistics Values in parentheses are for the highest of 20 resolution shells.

	Apo	AMP-bound
Wavelength (Å)	1.5418	1.5418
Space group	*P*2_1_2_1_2_1_	*P*2_1_2_1_2_1_
Unit-cell parameters (Å)	*a* = 52.72, *b* = 73.05, *c* = 75.01	*a* = 53.24, *b* = 73.18, *c* = 75.38
Resolution range (Å)	20–1.85 (1.90–1.85)	20–2.00 (2.05–2.00)
Mean *I*/σ(*I*)	19.6 (2.1)	16.4 (2.3)
*R*_merge_†	0.045 (0.498)	0.079 (0.650)
Completeness (%)	99.7 (99.6)	99.1 (92.9)
Multiplicity	5.0 (3.0)	6.6 (3.9)
No. of unique reflections	25330	20333

† *R*_merge_ = 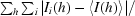 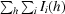.

**Table 3:** Refinement and model statistics Values in parentheses are for the highest of 20 resolution shells.

	Apo	AMP-bound
Resolution range (Å)	20–1.85 (1.90–1.85)	20–2.00 (2.05–2.00)
*R*_cryst_†	0.184	0.196
*R*_free_‡	0.236	0.265
R.m.s.d. bonds (Å)	0.018	0.018
R.m.s.d. angles (°)	1.59	1.61
Protein atoms	2188	2212
Nonprotein atoms	209	167
Mean *B* factor (Å^2^)	31.7	23.7
Residues in favored region	250 [95%]	253 [95%]
Residues in allowed region	8 [3.1%]	9 [3.4%]
Residues in disallowed region	4 [1.5%]	3 [1.1%]
*MolProbity*§ score [percentile]	1.62 [90th]	1.97 [76th]
PDB code	3gbz	3gc0

† *R*_cryst_ = 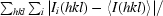 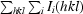. The free *R* factor was calculated using an equivalent equation as for *R*_cryst_ with 5% of the reflections that were omitted from the refinement.

‡ Chen *et al.* (2010 ▶)
